# A cell-targeted chemotherapeutic nanomedicine strategy for oral squamous cell carcinoma therapy

**DOI:** 10.1186/s12951-015-0116-2

**Published:** 2015-10-01

**Authors:** Zhi-Qi Wang, Kai Liu, Zhi-Jun Huo, Xiao-Chen Li, Min Wang, Ping Liu, Bo Pang, Shi-Jiang Wang

**Affiliations:** Department of Head and Neck Surgery, Shandong Cancer Hospital and Institute, Jinan, 250117 China; Department of Gastrointestinal Surgery, Shandong Cancer Hospital and Institute, Jinan, 250117 China; Department of Breast Disease Center, Shandong Cancer Hospital and Institute, Jinan, 250117 China; Department of Internal Medicine, Affiliated Hospital of Shandong Academy of Medical Sciences, Jinan, 250031 China; Department of Pathology, The Second People’s Hospital of Liaocheng, Linqing, 252600 Shandong China; Department of Pharmacy, Shandong Provincial Hospital Affiliated to Shandong University, Jinan, 250021 China; Department of Neurosurgery, Qilu Hospital, Shandong Univeristy, 107# Wenhua Xi Road, Jinan, 250012 China; Department of Radiation Oncology, Shandong Cancer Hospital and Institute, Jinan, 250117 Shandong China

**Keywords:** Oral squamous cell carcinoma, Cisplatin (CDDP), Polymeric micelles, Antitumor efficacy

## Abstract

**Background:**

Oral squamous cell carcinoma (OSCC) or cancers of oral cavity is one of the most common cancers worldwide with high rate of mortality and morbidity. At present, chemotherapy is one of the most effective treatments; however it often fails to meet the requirements in the clinical therapy. In the present study, we have successfully formulated ligand-decorated cancer-targeted CDDP-loaded PLGA-PEG/NR7 nanoparticles and demonstrated the feasibility of using NR7 peptide for targeted delivery, rapid intracellular uptake, and enhanced cytotoxic effect in receptor-overexpressed OSCC cancer cells.

**Results:**

Nanosized particles were formed and sustained release patterns were observed for PLGA/NR7 nanoparticles. Significantly higher cellular uptake was observed in HN6 OSCC cancer cells and superior anticancer effects are observed from the optimized targeted nanoparticles. Furthermore, Live/Dead assay showed a higher extent of red fluorescence was observed for the cells exposed with PLGA/NR7 than compared with non-targeted PLGA NP. The presence of the NR7-targeting moiety on the surface of PLGA carriers could allow the specific receptor-mediated internalization, enhanced cellular uptake, and higher cell killing potency. Especially, PLGA/NR7 NP exhibited a superior apoptosis effect in HN6 cancer cells with around ~45 % (early and late apoptotic stage) and ~59 % after 24 and 48 h incubation, respectively. It is apparent that the actively targeted micelles will deliver more anticancer agent to cancer cell than non-targeted one.

**Conclusion:**

Altogether, our results show the feasibility and promise of a cell-targeted anticancer nanomedicine strategy that can be effective for the treatment of oral squamous cell carcinoma. The present work might be of great importance to the further exploration of the potential application of PLGA/NR7 in the clinically relevant animal models.

## Background

Oral squamous cell carcinoma (OSCC) or cancers of oral cavity is one of the most common cancers worldwide, especially in developing nations like China [1]. OSCC become a critical healthcare problem with high rate of mortality and morbidity [2]. Squamous cell carcinoma of the oral cavity accounts for close to ~400,000 cases per year with a mortality rate of 50 % [3]. Present treatment options include surgery, radiotherapy, and conventional chemotherapy. Due to the specific location of anatomic structures (breathing and swallowing), surgical excision of tumor tissues in this region causes unnecessary damage to adjacent or underlying anatomical structures [4]. Whereas, radiation therapy may have long-term side effects to the healthy cells which are associated with brain, spinal cord, and saliva glands (such as xerostomia and osteoradionecrosis). At present, chemotherapy is one of the most effective treatments; however it often fails to meet the requirements in the clinical therapy. Generally, conventional chemotherapeutics drugs exhibit poor systemic stability, limited water solubility, unwanted drug-related side effects (bone marrow depression and nephrotoxicity), and relatively short half-life that prevent their further clinical application [5, 6]. Despite recent progress in the diagnosis of and therapeutic modalities for OSCC, overall survival rates have not improved and needs alternative therapeutic approaches.

In this regard, cis-Diaminedichloroplatinum (cisplatin, CDDP) is extensively used for the treatment of various cancers such as ovarian, testicular, colorectal, and oral squamous cancers [7, 8]. CDDP is recommended in multiple cancers owing to its strong synergistic anticancer effect either as single drug or in combination with other anticancer agents. CDDP kills cancer cell by inducing cross-linking of DNA by interfering with the cellular divisions and activates the apoptosis pathways [9]. Additionally, it suppresses the Bcl-2 protein in many cancer cells. Despite, its potential therapeutic effects, CDDP suffers from many serious side effects such as nephrotoxicity, neurotoxicity, gastrointestinal toxicity, hematological toxicity and ototoxicity [10, 11]. Therefore, effective controlled delivery systems have to be designed to target to oral cancer sites and to avoid the shortcomings of conventional chemotherapy treatments.

Recently, self-assembled polymeric nanoparticles, have received increased attention for their potential application as a drug delivery carrier in cancer therapeutics. The polymeric self-assembled nanoparticles offer some unique advantages including core–shell morphology, high loading capacity, site-specific drug delivery, and avoids unwanted side effects of administered drug. For this purpose, biodegradable polymer, poly(lactic-co-glycolic acid) (PLGA)-poly(ethylene) glycol (PEG) (PLGA-PEG) was selected due to its excellent systemic characteristics and biodegradability [12]. Several studies have reported that nanosized PLGA-PEG NP would effectively increase the intracellular concentration of anticancer drugs by enhancing the blood circulation time of nanocarriers and avoids the reticuloendothelial system (RES) mediated clearance [13]. Moreover, polymeric nanoparticles with active targeting moiety will increase the target specificity. The epidermal growth factor (EGF) receptor is recognized as an important target for the development of treatment for cancer [14]. EGF receptor is highly expressed in human epithelial cancer cells such as OSCC. Several EGF-targeting therapeutic agents such as cetuximab and erlotinib have already been approved by the USFDA. In this line, NR7 peptide (NSVRGSR) which is based on alignment of the tripeptide motif with the EGF binding domain was selected [15]. Moreover, NR7 is the only peptide which not only appeared twice between these EGF receptor ligands but also contained tripeptide motifs relative to the mature EGF domain [16]. We expected that when NR7-conjugated delivery system is administered, it will enhance the overall chemotherapeutic efficiency in cancers.

Herein, the main aim of present study was to develop a CDDP-loaded delivery system to target the oral squamous cell carcinoma. For this purpose, PEG-PLGA polymer was synthesized and conjugated with NR7 peptide. The CDDP-loaded PLGA NP was prepared by solvent-evaporation method and evaluated in terms of size, shape, in vitro release study (physicochemical characterizations). Further, target specificity was investigated in the in vitro conditions by means of cellular uptake of targeted and non-targeted NP in OSCC cancer cells. Cytotoxic effect of targeted and non-targeted NP was investigated by MTT assay, live-dead assay, and apoptosis analysis.

## Results and discussion

**Fig. 1:**
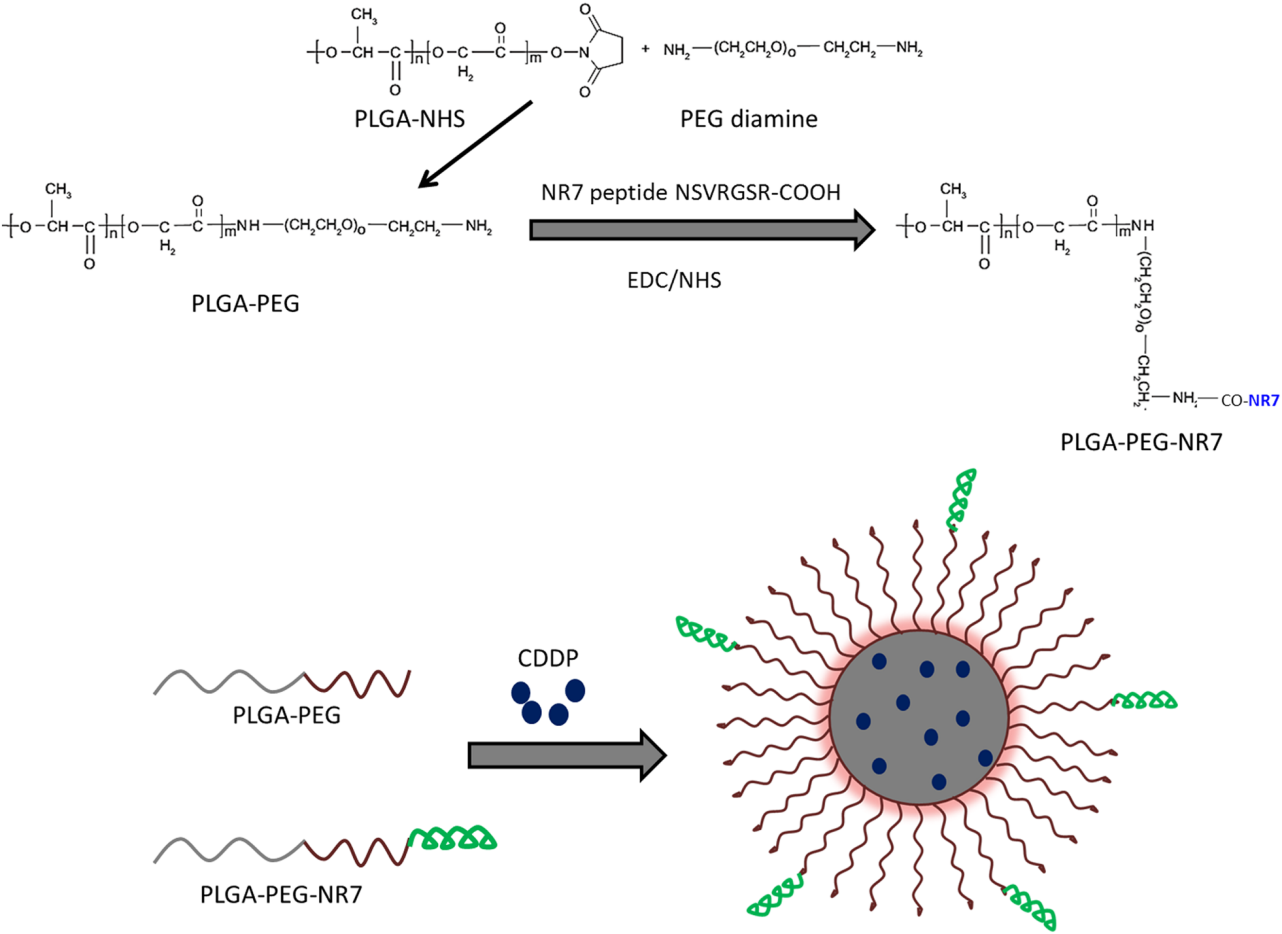
Schematic illustration of synthesis of PLGA-PEG and PLGA-PEG-NR7. The self-assembly of cisplatin (CDDP) with PLGA-PEG-NR7 block copolymer into a polymeric micelles is presented

Squamous cell carcinoma of the oral cavity accounts for close to ~ 400,000 cases per year with a mortality rate of 50 %. At present, chemotherapy is one of the most effective treatments; however it often fails to meet the requirements in the clinical therapy. Generally, conventional chemotherapeutics drugs exhibit poor systemic stability, limited water solubility, unwanted drug-related side effects (bone marrow depression and nephrotoxicity), and relatively short half-life that prevent their further clinical application. Cis-Diaminedichloroplatinum (cisplatin, CDDP) is one such drug which is extensively used for the treatment of various cancers such as ovarian, testicular, colorectal, and oral squamous cancers, however it suffers from many serious side effects. Studies have shown that nanocarriers caused better selective accumulation of CDDP in tumors while lessening its distribution in normal tissue. Therefore, biodegradable polymer, poly(lactic-co-glycolic acid) (PLGA)-poly(ethylene) glycol (PEG) (PLGA-PEG) based self-assembled polymeric micelles was designed in the present study. The hydrophilic poly(ethylene glycol) shell layer enables the particles to circulate for long time in the blood compartment which will facilitate its preferential accumulation in the tumor tissues. Furthermore, NR7 peptide (NSVRGSR) which is based on alignment of the tripeptide motif with the EGF binding domain was selected to actively target the anticancer drug to the specific cancer site (Fig. 1). We expected that when NR7-conjugated delivery system is administered, it will enhance the overall chemotherapeutic efficiency in cancers.

In this study, PLGA-PEG was conjugated with NR7 peptide to increase the tumor specificity. The so-formed block polymer exhibited a critical micellar concentration of 3.3 × 10^−8^ mol/L. Our results are consistent or better than the reported values. It has been earlier reported that micelles with a CMC of 8 × 10^−7^ mol/L was stable in the blood circulation. In this regard, our micelles which showed much lower CMC value is expected to show a remarkable systemic performance.

### Physicochemical characterization of nanoparticles

**Fig. 2:**
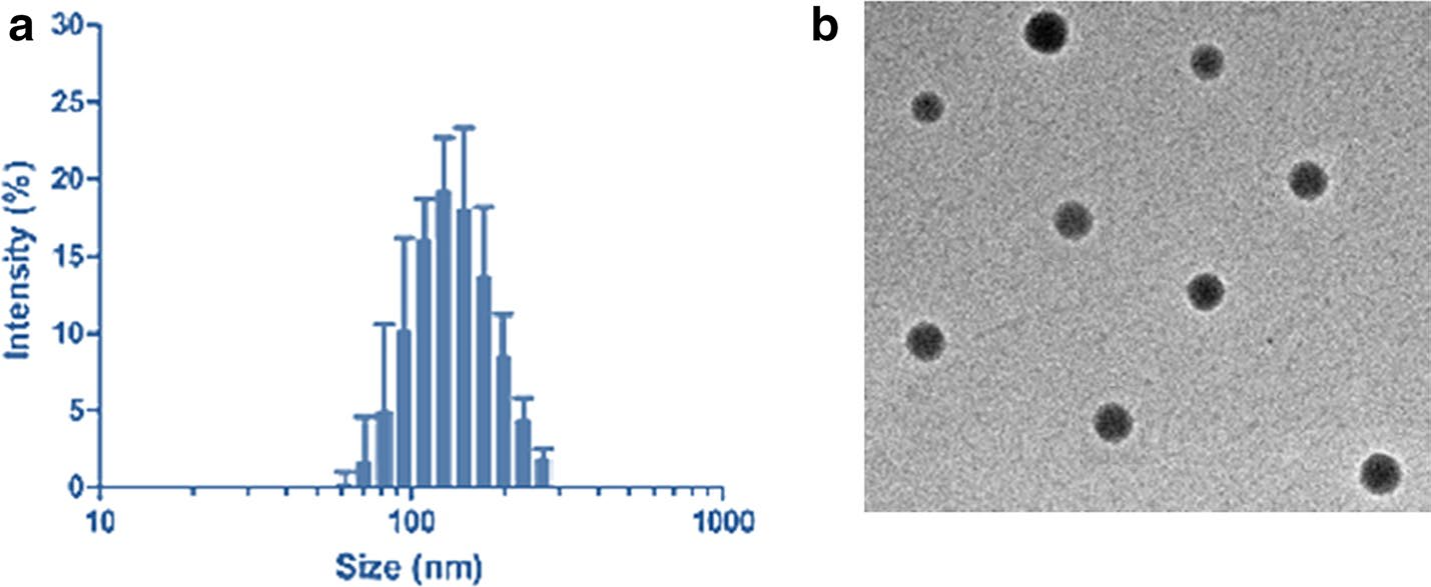
**a** Particle size distribution of CDDP-loaded PLGA-PEG-NR7 nanoparticles. **b** TEM image of PD/IFS

The CDDP-loaded PLGA NPs were formed by the self-assembly of drug in the hydrophobic core of the polymeric micelles. After being dissolved in DMSO and dialyzed against water, CDDP was able to spontaneously self-assemble into micelles with hydrophobic PLGA core and hydrophilic PEG outer shell. The resulting NPs were expected to be nanometre range. The particle size and size distributions of NP were evaluate by dynamic light scattering technique (DLS). The average size of drug-loaded PLGA NP was around ~100 nm while a slight increment in size was observed for PLGA/NR7 NP (~135 nm) (Fig. 2a). The slight increase in size could be attributed to the presence of NR7 peptide on the surface of the nanoparticles. Nevertheless, both the NP system showed an excellent polydispersity index of PDI ~ 0.15. Despite the increase in the overall size, resulting size was in accordance with literature for cancer drug delivery applications. It has been reported that micelles with an average size between 50- and 150-nm-diameter ranges, could easily avoid rapid renal clearance or leakage through normal vasculature, yet allow EPR-mediated accumulation through tumor-associated leaky vasculature [17, 18].

The morphology of optimized PLGA/NR7 NP was evaluated by means of transmission electron microscopy (TEM) (Fig. 2b). The PLGA/NR7 NP showed a nanosized particle with clear spherical morphology. The particles were uniformly distributed on the carbon-coated copper grid suggesting the success of the formulation technique. A darker core represents the hydrophobic PLGA core while a shallow outer layer (greyish) could be the PEG shell. Such a nanosized particle with spherical morphology is expected to increase the systemic performance and anticancer effect.

### In vitro drug release study

**Fig. 3:**
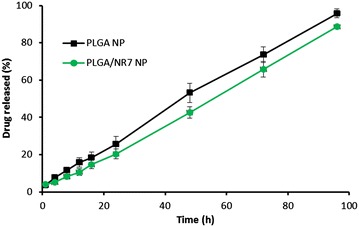
The release profile of CDDP from PLGA NP and PLGA/NR7. The release study was performed in phosphate buffered saline at 37 °C. The study was carried out for 96 h

The drug release profile of PLGA NP and PLGA/NR7 NP was investigated by dialysis method under simulated physiological condition (PBS, pH 7.4) at 37 °C. The results revealed a constant and controlled release of CDDP from the nanoparticulate systems (Fig. 3). Throughout the study period (96 h), CDDP was released in a controlled manner with no burst release pattern or bi-phasic release pattern. Lack of burst release pattern indicates that the drug was stably loaded in the hydrophobic core of the polymeric micelles. Importantly, presence of NR7 peptide on the surface of NP did not affect the release of anticancer drug. A slight decrease in the release rate of CDDP from PLGA/NR7 NP might be attributed to the aforementioned reason. Overall, ~85 % of drug released from PLGA NP and ~80 % of drug released from PLGA/NR7 NP. The release study clearly showed that PLGA NP could effectively protect the drug in the systemic circulation which will allow the preferential accumulation of NP in the tumor tissues. It could be expected that release will be faster in the tumor acidic pH owing to the intracellular compartments (endosomes and lysosomes).

### In vitro cellular uptake study

**Fig. 4:**
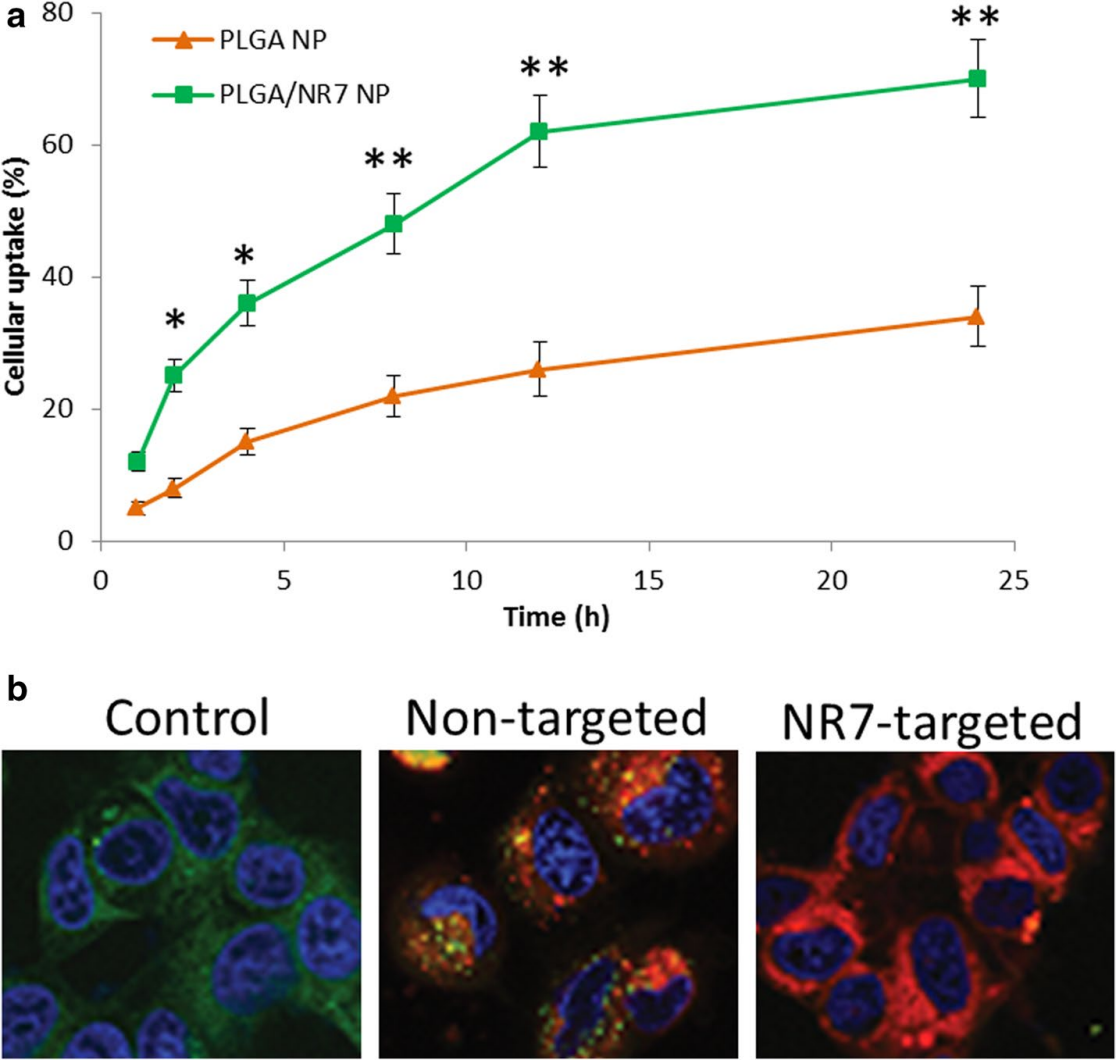
**a** Intracellular uptake of PLGA NP and PLGA/NR7 NP in HN6 squamous cell carcinoma. Rhodamine B was used as a fluorescent dye. The uptake is shown as a percentage of total amounts of NP (dye) incubated with the cancer cells. **b** Representative confocal microscopy images of targeted and non-targeted NP in HN6 cancer cells. The cells are stained with Lysotracker lysosomal stain and DAPI was used to stain the nucleus. **p < 0.01 and *p < 005 is the statistical difference between the cellular uptake of two formulations

Quantitative and fluorescent studies were performed to investigate the binding affinity of targeted and non-targeted polymeric micelles. For this purpose, PLGA NP was loaded with Rho B fluorescent dye instead of CDDP. As seen from Fig. 4a, cells incubated with PLGA and PLGA/NR7 NP showed appreciable intracellular uptake. Notably, NR7 conjugated polymeric nanoparticle showed significantly (*p* < 0.01) higher cellular uptake than that of non-targeted PLGA NP. The influence of targeting ligand on the NP surface was visible immediately after 1 h of incubation, wherein PLGA/NR7 NP exhibited more than 20 % of cellular internalization compared to <10 % for non-targeted NP. The trend continued up to 24 h. Two different phase of cellular uptake was observed. First, steady or faster rate of cell uptake was seen in first 12 h of study, followed by a relatively slower cellular uptake (of less than 10 %). Overall, PLGA/NR7 NP showed ~70 % of NP uptake comparing to meagre ~34 % for PLGA NP. From the result, it is apparent that the actively targeted micelles will deliver more anticancer agent to cancer cell than non-targeted one. These data suggest a role for receptor-mediated internalization of the actively targeted PLGA/NR7 NP.

The fluorescence microscopy images further supported the quantitative estimated for NP uptake. For this purpose, lysosome was stained with Lysotracker Green and the nuclear region was stained with DAPI. As seen from Fig. 4b, PLGA/NR7 NP treated cells showed remarkably high fluorescence intensity (70 %) than compared to PLGA NP treated cells (28 %). The difference between cellular internalization of two groups could be the mechanism of cellular uptake. A typical endocytosis-mediated active uptake could be the main reason behind the high uptake of NR7 peptide conjugated NP group, whereas normal membrane based passive processes might be involved in non-targeted NP [19]. This resulted in low colocalization of red fluorescence with lysosomal (green) fluorescence. These data suggest that without active NR7 targeting, the intracellular uptake of NP would be significantly less. Similar mechanisms have been recently reported for a number of ligand decorated drug-loaded block co-polymer micelle formulations targeted to relevant cancer biomarkers like EGFR, folate receptor, and integrin αVβ3. The nanoformulation is expected to destabilize and disassemble in the lysosomal compartment to allow CDDP release which will then travel to nucleus and perinuceus region and enhance the anticancer efficacy [20]. These results provide the rationale that upon in vivo administration; these nanoformulations may allow targeted CDDP delivery, tumor-selective accumulation, and rapid intracellular uptake.

### In vitro cytotoxicity assay

**Fig. 5:**
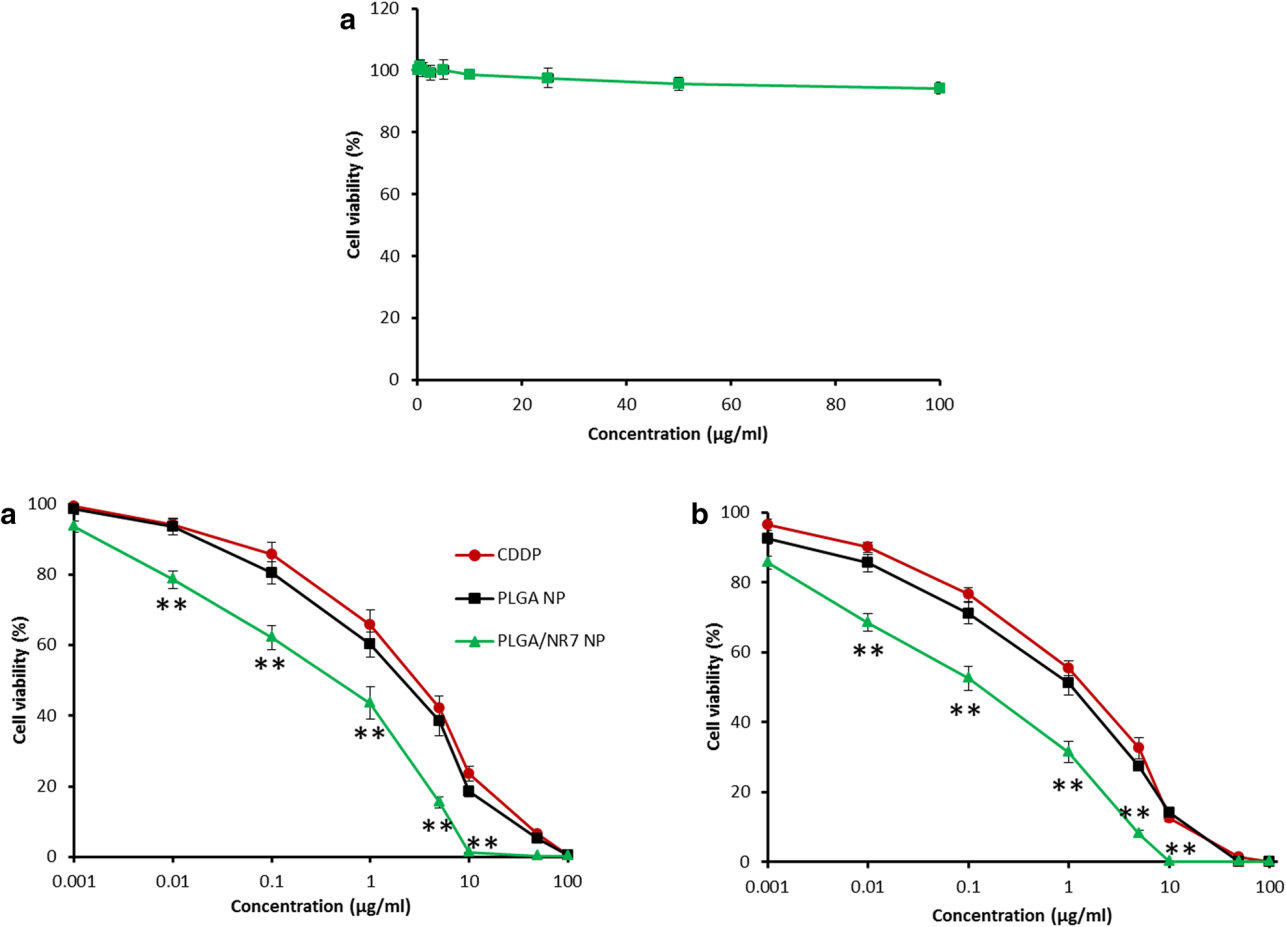
**a** Cytotoxicity assay of blank nanoparticles in HN6 cancer cells. **b** Cytotoxicity analysis of free CDDP, PLGA NP, and PLGA/NR7 NP in HN6 cancer cells. Different amounts of formulations were added from 0.001 to 100 µg/ml to each well and incubated for 24 and 48 h. Survival rate of HN6 cell was determined by MTT assay. Data were obtained from three independent triplicate experiments and were presented as mean ± S.D. **p < 0.01 is the statistical difference between the cytotoxicity of PLGA/NR7 and CDDP

The anticancer efficacy of free CDDP, PLGA NP, and PLGA/NR7 NP was evaluated on squamous cell carcinoma (HN6) using MTT assay. The cells were treated with individual formulations at various concentrations and incubated for 24 and 48 h, respectively. First, blank nanoparticle was incubated in HN6 cells and noted its cytotoxic effect. As seen from Fig. 5a, blank polymer did not induce any appreciable toxicity and the cell viability remained more than 95 % throughout all the concentrations tested. The high cell viability of blank polymer indicates its excellent biocompatibility and would be suitable for the systemic administration or cancer targeting.

The free drug as well as drug-loaded formulations exhibited a dose-dependent and time-dependent cytotoxicity on HN6 cancer cells (Fig. 5b, c). Specifically, PLGA/NR7 NP showed a significantly (*p* < 0.01) higher cytotoxic effect than compared to either free CDDP or PLGA NP. The results were consistent with cellular uptake study wherein NR7 active targeting moiety conjugated group showed an enhanced cellular uptake. The presence of the NR7-targeting moiety on the surface of PLGA carriers could allow the specific receptor-mediated internalization, enhanced cellular uptake, and higher cell killing potency. Increased anticancer activity with the increase in the incubation of formulations could be attributed to higher intracellular concentration of anticancer drugs [21]. The doses for cytotoxic effect were determined based on IC50 values of these NPs. The IC50 value of free CDDP, PLGA NP, and PLGA/NR7 NP were observed to be 4.94, 2.61, and 0.75 µg/ml, respectively after 24 h of incubation. This is in accordance with the fact that at shorter time periods, for the NR7-targeted PLGA nanoformulation, a higher amount of intracellular uptake of CDDP occurs via receptor-mediated mechanism, compared to the time-dependent passive uptake of the nontargeted formulation. This results in the superior anticancer effect of the NR7 conjugated formulations [22].

### Live/dead assay

**Fig. 6:**
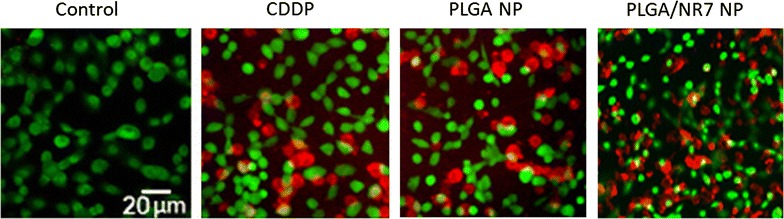
The cytotoxicity potential of free CDDP, PLGA NP, and PLGA/NR7 NP were evaluated by Live/Dead assay. Cells were stained with a live/dead cell viability assay (Invitrogen). This assay uses two fluorescent probes, calcein AM and ethidium homodimer-1

The cytotoxicity assay was further confirmed by Live/Dead assay. The cells were treated with respective formulations and then incubated with Calcein AM and Ethidium bromide dye as representative live and dead cell markers. As seen from Fig. 6, higher extent of red fluorescence (68 %) was observed for the cells exposed with PLGA/NR7 than compared with non-targeted PLGA NP (39 %). Consistent with cytotoxicity assay, free CDDP showed 40 % more viable cells (green fluorescence) and the red fluorescence was significantly decreased. Whereas extent of red and green fluorescence is an indication of dead cells and live cells, respectively. Despite the cytotoxic effect of PLGA/NR7 carrier, slight presence of green cells suggest that drug release from the nanoparticle in a sustained manner and therefore kills in a time-dependent manner. Generally, Calcein AM can easily enter cells by diffusion and it is converted to Calcein by the intracellular esterase which stains the live cells green. The damaged or dead cells are stained red with Ethidium bromide.

### Cell apoptosis assay

**Fig. 7:**
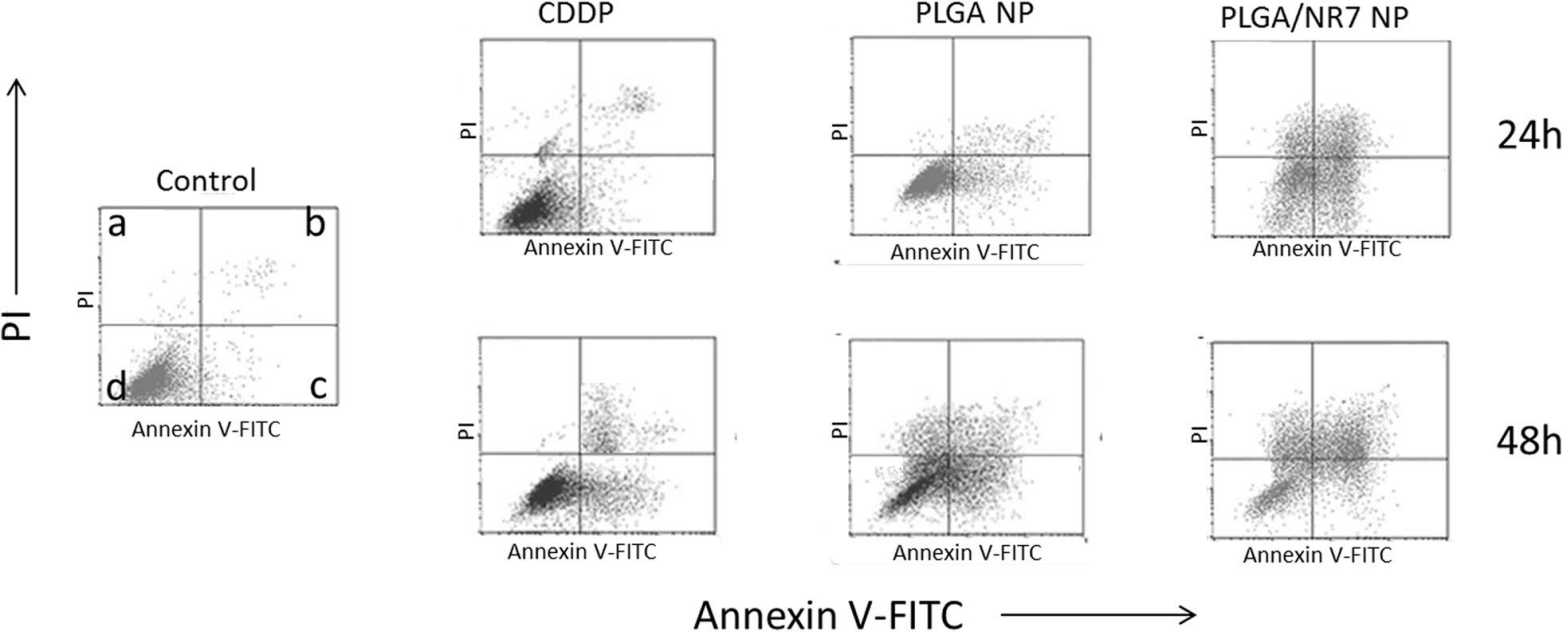
Annexin V-FITC FACS apoptosis analysis of HN6 cancer cells after treatment with free CDDP, PLGA NP, and PLGA/NR7 NP for 24 and 48h, respectively. **a** indicates PI positive cells (necrosis), **b** represents late apoptosis (PI and Annexin positive cells), **c** represents healthy cells (PI and Annexin negative cells), **d** early apoptosis (Annexin positive cells)

Annexin V-FITC/PI staining assay was used to investigate the mode of cell death and apoptosis for free CDDP, PLGA NP, and PLGA/NR7 NP treated groups. Annexin V/FITC detects the phosphatidylserine externalization which characterizes early apoptotic events. Cancer cell death can be programmed (apoptosis) or non-programmed (necrosis). The apoptosis-inducing effect of formulations was evaluated by counting the early apoptosis and late apoptosis. The results clearly showed a remarkable apoptosis effect of all the formulations (Fig. 7). Especially, PLGA/NR7 NP exhibited a superior apoptosis effect in HN6 cancer cells with around ~45 % (early and late apoptotic stage) and ~59 % after 24 and 48 h incubation, respectively. One could easily see that at 24 h, early apoptosis was predominant however with the increase in the incubation time; cells migrate to late apoptosis stage, which is consistent with many published reports. On the other hand, PLGA NP showed around ~26 and ~43 % apoptosis after respective time interval. The result was consistent with the cellular uptake analysis and MTT-based cytotoxicity assay. The plausible reason for such superior anticancer activity by PLGA/NR7 nanoparticles could be attributed to the in vitro release profile and cell uptake studies. The results indicate that the high cytotoxicity induced by PLGA/NR7 NP can mainly be attributed to apoptosis induction which was in turn due to the high cellular internalization of targeted nanoparticles.

### Conclusion

In conclusion, we have successfully formulated ligand-decorated cancer-targeted CDDP-loaded PLGA-PEG/NR7 nanoparticles and demonstrated the feasibility of using NR7 peptide for targeted delivery, rapid intracellular uptake, and enhanced cytotoxic effect in receptor-overexpressed OSCC cancer cells. Nanosized particles were formed and sustained release patterns were observed for PLGA/NR7 nanoparticles. Significantly higher cellular uptake was observed in HN6 OSCC cancer cells and superior anticancer effects are observed from the optimized targeted nanoparticles. Furthermore, Live/Dead assay showed a higher extent of red fluorescence was observed for the cells exposed with PLGA/NR7 than compared with non-targeted PLGA NP. The presence of the NR7-targeting moiety on the surface of PLGA carriers could allow the specific receptor-mediated internalization, enhanced cellular uptake, and higher cell killing potency. Especially, PLGA/NR7 NP exhibited a superior apoptosis effect in HN6 cancer cells with around ~45 % (early and late apoptotic stage) and ~59 % after 24 and 48 h incubation, respectively. It is apparent that the actively targeted micelles will deliver more anticancer agent to cancer cell than non-targeted one. Altogether, our results show the feasibility and promise of a cell-targeted anticancer nanomedicine strategy that can be effective for the treatment of oral squamous cell carcinoma.

## Materials and methods

### Materials

PLGA ([η] = 0.32–0.44 dL/g; 50:50) and CDDP was purchased from Sigma-Aldrich (China). Cisplatin, PEG-diamine (Mn ~ 2000) was purchased from Laysan Bio Inc (Arab, AL). 1-ethyl-3 (3-dimethylaminopropyl) carbodimide (EDC) was purchased from Sigma Aldrich, China. All other chemicals were of reagent grade and used without further purifications.

### Fabrication of PLGA-PEG block copolymer

PLGA-PEG was fabricated using carbodiimide coupling reaction. Briefly, 10 g of PLGA was activated by dissolving in DMSO and followed by the addition of EDC and NHS and the mixture was allowed to react for 15 h under constant nitrogen atmosphere. PLGA-NHS was collected by the addition of cold ether and filtered. The PLGA-PEG was formed by dissolving both PLGA-NHS and PEG diamine at a ratio of 30:1 in DMSO and allowed to react for 24 h under constant nitrogen atmosphere at room temperature. The final product PLGA-PEG-NH2 was precipitated by excessive acetonitrile and washed with ice cold methanol. The unreacted PEG-diamine was separated by dialysis method and dried under vacuum.

PLGA-PEG-NR7 was prepared by chemical conjugation of carboxylic group of peptide with the amino group of PLGA-PEG copolymer in the presence of carbodiimide hydrochloride (EDC) and N-hydroxysuccinimide (NHS). The PLGA-PEG and NR7 peptide was mixed at a ratio of 10:1 and allowed to react at room temperature for 3 h. The final product was precipitated, centrifuged, and freeze dried.

### Preparation of CDDP-loaded polymeric nanoparticles

CDDP and PLGA-PEG was dissolved in acetone/dichloromethane (1/1 v/v) mixture at a ratio of 5:1. This organic solution was slowly added to 10 mL of 0.25 % polyvinyl alcohol and the solution was immediately sonicated (3 min) and mechanically stirred at room temperature for 2 h. The drug-loaded nanoparticles (NP) was collected by centrifuging at high speed and re-suspended in ultra-pure water. The CDDP-loaded PLGA-PEG NP (PLGA/NR7) was stored at 4 °C until further analysis.

### Live/dead assay

Live/dead assay was performed using a Live/dead cell viability assay Kit (Invitrogen). Calcein AM and ethidium homodimer-1 was identified as markers for live and dead cell, respectively. Nonfluorescent calcein-AM is hydrolysed by live cell into green fluorescent calcein, whereas ethidium homodimer-1 can only pass through dead cells membrane. For this purpose, cells were seeded in a 12-well plate and incubated for 24 h. The cells were treated with respective formulations and further incubated for 24 h. Subsequently, the cells were rinsed twice with PBS before the fluorochromes were added and incubated for 45 min. Fluorescence images were then taken using a Floid Cell Imaging Station (Molecular Probes Life Technology, France).

### Particle size and size distribution analysis

The average particle size and size distribution analysis was performed using a Zetasizer Nano-S90 (Malvern Instruments, Malvern, UK) and a 633 nm He–Ne laser beam at a fixed scattering angle of 90°. A dilute solution of NP was used to analyse the particle size. The experiments were performed in triplicates.

### Transmission electron microscopy

The morphology and size of particles were observed by using the HITACHI 7650 transmission electron microscope (TEM) from Hitachi, Ltd (Kyoto, Japan). Before the examinations, NP dispersion was diluted many times with ultra-pure water. The aqueous solution was dropped on the carbon coated copper grid and counter stained with 2 % phosphotungistic acid. The samples were dried using an infrared lamp and viewed under TEM.

### Drug loading

The drug encapsulation efficiency (EE %) was expressed as the percentage of the amount of CDDP encapsulated in the nanoparticles to the total amount of CDDP initially used. The drug loading (DL %) was expressed as the percentage of the amount of CDDP encapsulated in the nanoparticles to the total amount of nanoparticles initially used. The loading efficiency and loading capacity was determined as follows. In brief, 10 mg of lyophilized NP was dissolved in 5 ml of DMSO and sonicated for 15 min. The organic solution was centrifuged and the supernatant was used to calculate the amount of drug loaded. The supernatants were then determined using the ICP-MS with the following experimental parameters: plasma flow (15 L/min), auxiliary flow (1.5 L/min), radio frequency power (1300 W), sampling depth (8.4 mm), atomizing chamber temperature (2 °C), and nebulizer flow (1.0 L/min).

### Drug release study

The CDDP release from the NP system was determined using a dialysis method. Briefly, 30 mg of PLGA NP lyophilized powder was dissolved in 1 ml of water and sealed in a dialysis tube. The dialysis tube was in turn placed in a 50 ml of Falcon tube containing 25 ml of release media. Selective release media including phosphate buffered saline (PBS, pH 7.4) was used. The sampling was done at specific time points such as 1,2,4.6,8,10,12,24,48,72,96,120 h. At each sampling point, 1 ml of release sample was withdrawn and replaced with equal volume of fresh media. The released IFS content in the released medium was determined as reported previously. The CDDP encapsulated in nanoparticles was measured according to the previous method by using the HITACHI P-4010 inductively coupled plasma mass spectrometry (ICP-MS) from Hitachi Ltd (Kyoto, Japan).

### Cytotoxicity assay

The human OSCC cell lines HN-6 was cultured in DMEM supplied with 10 % FBS and antibiotics (50 units/mL penicillin and 50 units/mL streptomycin) at 37 °C in a humidified atmosphere containing 5 % CO2. The cytotoxicity potential of individual formulations were evaluated by means of MTT colorimetric assay. Cells (1 × 104 per well) were seeded in a flat-bottomed 96 well plate and incubated at 37 °C and in 5 % CO_2_. Cells were exposed to blank NPS and drug-loaded formulations at various dose concentrations. The cells were incubated for 24 and 48 h, respectively. Cells were then treated with MTT reagent (20 μL/well volume from 5 mg/mL solution in PBS) for 3 h at 37 °C. DMSO (100 μL) was added to each well to dissolve the formazan crystals. The optical density (OD) was recorded at 570 nm in a microplate reader, and percentage of residual cell viability was determined. The cell cytotoxicity of different formulations is defined as the relative viability, which is the ratio of the number of live cells to that of the control cells (100 %).

### Fluorescence microscopy

The cellular uptake efficiency of PLGA NP and PLGA/NR7 NP in OSCC was evaluated by fluorescence and confocal laser scanning microscopy. HN-6 cells were seeded into 24-well plates (5 × 10^4^ cells/well) and incubated for 24 h. The medium was then replaced with fresh DMEM culture medium containing PLGA NP and PLGA/NR7 NP. For the cellular uptake studies, CDDP was replaced with Rho B as a fluorescent marker. The cells were incubated for various time points up to 24 h. At each time points, cells were washed; extracted and cellular uptake was calculated in terms of extent of fluorescent intensity.

The cellular internalization process was further confirmed by confocal laser scanning microscopy. HN-6 cells were seeded into 6-well plates (1 × 10^6^ cells/well) and incubated for 24 h. Cells were treated with PLGA NP and PLGA/NR7 NP, respectively. The cells were then washed with PBS and fixed with 4 % paraformaldehyde for 15 min at room temperature, and the slides were rinsed with cold PBS three times. The cells were observed using fluorescence microscope (Olympus Bx60).

### Apoptosis assay

Cells (1 × 105 per well) were seeded in a flat-bottomed 12 well plate and incubated at 37 °C and in 5 % CO2. Cells were exposed to different drug-loaded formulations at various dose concentrations. The cells were incubated for 24 h and were harvested and washed with PBS after the treatment. The cells were first incubated with 100 μL of binding buffer and stained with 5 μL of Annexin V-FITC and 5 μL of propidium iodide (PI) from kit according to manufacturer’s instruction (BD Bioscience, China) at room temperature for 15 min in the dark. The cells were then analyzed by flow cytometry (BD LSR II Analyzer), and 10,000 cells were counted.

### Statistical analysis

Statistical analyses were carried out using SigmaStat version 2.0 (SPSS, Inc, Chicago, IL, USA). Unless stated otherwise, all experiments were performed in triplicate, and results are presented as mean values ± standard deviation. ANOVA testing was employed to determine the statistical significance with *p* < 0.05.

## Abbreviations

CDDP: Cisplatin
OSCC: oral squamous cell carcinoma
PLGA: poly (lactic-b-glycolic) acid
EGF: epidermal growth factor
EGFR: epidermal growth factor receptor

## References

[1] Lopes CF, de Angelis BB, Prudente HM, de Souza BV, Cardoso SV, de Azambuja Ribeiro RI (2012). Concomitant consumption of marijuana, alcohol and tobacco in oral squamous cell carcinoma development and progression: recent advances and challenges. Arch Oral Biol.

[2] Kademani D, Bell RB, Brian (2008). Oral and maxillofacial surgeons treating oral cancer: a preliminary report from the American Association of Oral and Maxillofacial Surgeons Task Force on Oral Cancer. J Oral Maxillofac Surg.

[3] Woolgar JA, Triantafyllou A (2005). A histopathological appraisal of surgical margins in oral and oropharyngeal cancer resection specimens. Oral Oncol.

[4] Rosenthal EL, Kulbersh BD, Duncan RD (2006). In vivo detection of head and neck cancer orthotopic xenografts by immunofluorescence. Laryngoscope.

[5] Chen SF, Nieh S, Jao SW (2012). Quercetin suppresses drug-resistant spheres via the p38 MAPK − Hsp27 apoptotic pathway in oral cancer cells. PLoS One.

[6] Zhang P, Zhang Z, Zhou X, Qiu W, Chen F, Chen W (2006). Identification of genes associated with cisplatin resistance in human oral squamous cell carcinoma cell line. BMC Cancer.

[7] Bosl GJ, Motzer RJ (1997). Testicular germ-cell cancer. N Engl J Med.

[8] Dhar S, Kolishetti N, Lippard SJ, Farokhzad OC (2011). Targeted delivery of a cisplatin prodrug for safer and more effective prostate cancer therapy in vivo. Proc Natl Acad Sci USA.

[9] Galanski M, Jakupec MA, Keppler BK (2005). Update of the preclinical situation of anticancer platinum complexes: novel design strategies and innovative analytical approaches. Curr Med Chem.

[10] Aranya I, Safirsteina RL (2003). Cisplatin nephrotoxicity. Semin Nephrol.

[11] Brillet G, Deray G, Jacquiaud C, Mignot L, Bunker D, Meillet D, Raymond F, Jacobs C (1994). Long-term renal effect of cisplatin in man. Am J Nephrol.

[12] Avgoustakis K (2004). Pegylated poly(lactide) and poly(lactide-co-glycolide) nanoparticles: preparation, properties and possible applications in drug delivery. Curr Drug Deliv.

[13] Betancourt T, Byrne JD, Sunaryo N (2009). PEGylation strategies for active targeting of PLA/PLGA nanoparticles. J Biomed Mater Res A..

[14] Pal SK, Figlin RA, Reckamp K (2010). Targeted therapies for non-small cell lung cancer: an evolving landscape. Mol Cancer Ther.

[15] Kolonin MG, Bover L, Sun J (2006). Ligand-directed surface profiling of human cancer cells with combinatorial peptide libraries. Cancer Res.

[16] Harris RC, Chung E, Coffey RJ (2003). EGF receptor ligands. Exp Cell Res.

[17] Master AM, Qi Y (2012). EGFR-mediated intracellular delivery of Pc 4 nanoformulation for targeted photodynamic therapy of cancer: in vitro studies. Nanomed Nanotech Biol Med.

[18] Ramasamy T, Tran TH, Choi JY (2014). Layer-by-layer coated lipid-polymer hybrid nanoparticles designed for use in anticancer drug delivery. Carbohydr Polym.

[19] Savic R, Eisenberg A, Maysinger D (2006). Block copolymer micelles as delivery vehicles of hydrophobic drugs: micelle-cell interactions. J Drug Target.

[20] Allen C, Yu Y, Eisenberg A, Maysinger D (1999). Cellular internalization of PCL(20)-b-PEO(44) block copolymer micelles. Biochim Biophys Acta.

[21] Xiong XB, Mahmud A, Uludag H, Lavasanifar A (2007). Conjugation of arginine–glycine–aspartic acid peptides to poly(ethylene oxide)-bpoly(epsilon-caprolactone) micelles for enhance intracellular drug delivery to metastatic tumor cells. Biomacromolecules.

[22] Zeng F, Lee H, Allen C (2006). Epidermal growth factor–conjugated poly(ethylene glycol)-block-poly(delta-valerolactone) copolymer micelles for targeted delivery of chemotherapeutics. Bioconjug Chem.

